# MicroRNAs: Novel Players in the Dialogue between Pancreatic Islets and Immune System in Autoimmune Diabetes

**DOI:** 10.1155/2015/749734

**Published:** 2015-08-03

**Authors:** Giuliana Ventriglia, Laura Nigi, Guido Sebastiani, Francesco Dotta

**Affiliations:** ^1^Diabetes Unit, Department of Medicine, Surgery and Neurosciences, University of Siena, 53100 Siena, Italy; ^2^Fondazione Umberto Di Mario ONLUS, c/o Toscana Life Science Park, 53100 Siena, Italy

## Abstract

MicroRNAs are small noncoding RNA molecules that regulate gene expression in all cell types. Therefore, these tiny noncoding RNA molecules are involved in a wide range of biological processes, exerting functional effects at cellular, tissue, and organ level. In pancreatic islets of Langerhans, including beta-cells, microRNAs are involved in cell differentiation as well as in insulin secretion, while in immune cells they have been shown to play pivotal roles in development, activation, and response to antigens. Indeed, it is not surprising that microRNA alterations can lead to the development of several diseases, including type 1 diabetes (T1D). Type 1 diabetes is the result of a selective autoimmune destruction of insulin-producing beta-cells, characterized by islet inflammation (insulitis), which leads to chronic hyperglycemia. Given the growing importance of microRNA in the pathophysiology of T1D, the aim of this review is to summarize the most recent data on the potential involvement of microRNAs in autoimmune diabetes. Specifically, we will focus on three different aspects: (i) microRNAs as regulators of immune homeostasis in autoimmune diabetes; (ii) microRNA expression in pancreatic islet inflammation; (iii) microRNAs as players in the dialogue between the immune system and pancreatic endocrine cells.

## 1. Introduction

Type 1 diabetes (T1D) is a chronic autoimmune disease characterized by the selective destruction of insulin-producing beta-cells by the immune system. In early disease stages, islets of Langerhans are characterized by insulitis, an inflammatory process mediated by T cells (both CD8+ and CD4+ lymphocytes), B-lymphocytes, NK cells, and macrophages. The immune infiltrate has a pivotal role in beta-cell demise; however, the exact mechanisms involved in the dialogue between pancreatic islets and immune infiltrating cells are still under investigation [1].

Growing evidence indicates that microRNAs (miRNA), short RNA molecules involved in post-transcriptional repression, play a crucial role both in pancreatic beta-cell biology and in immune cell homeostasis. Indeed, miRNA alterations have been reported in murine beta-cells when settled in a proinflammatory environment, which mimics the* in vivo* inflammatory milieu induced by islet-infiltrating cells [2]. In addition, miRNAs alterations have also been reported in circulating immune cells in type 1 diabetic subjects [3, 4].

miRNAs represent a class of evolutionary conserved small (18–24 nucleotides), endogenous, single stranded, noncoding RNA molecules, which are important regulators of gene expression. miRNAs influence RNA stability and translational efficiency by targeting the 3′ untranslated region (UTR) of messenger RNA, leading to its degradation or to inhibition of protein translation. The human genome encodes 1881 miRNA precursors generating more than 2000 mature miRNAs [5]. Since their first discovery in 1993, miRNAs have now been recognized as key players in a wide range of biological processes such as differentiation, proliferation, ageing, and cell death.

The various stages of miRNAs biogenesis occur both in the nucleus and in the cytoplasm, where their maturation is tightly controlled by several enzymes. Among them, two are of major importance: Drosha-DGCR8 in the nucleus and Dicer in the cytoplasm. Dicer is the final enzyme, which generates mature miRNAs, which in turn can be loaded into the RISC (RNA Induced Silencing Complex) assembly, thus mediating the functional role of miRNAs. An important aspect of miRNAs biology is represented by the complexity of post-transcriptional gene expression controlling network. Indeed, each miRNA can potentially regulate the expression of several genes, while, on the other hand, a single mRNA can be targeted by numerous miRNAs [6]. Therefore it is not surprising that the presence of miRNAs is strictly necessary for the regulation of gene expression and, consequently, for the homeostasis of whole cellular processes.

In this review we will specifically focus on the role of miRNAs in three important aspects of autoimmune diabetes etiology:

- The role of miRNAs in immune homeostasis and the influence of miRNA alterations on the development of autoimmune diabetes.
- miRNAs role in the response of pancreatic islets and/or of beta-cells to immune-mediated stress.
- Secreted miRNAs as a mechanism of islet-immune cells dialogue in autoimmune diabetes.

Importantly, all these three different faces of autoimmune diabetes are strictly linked among each other, thus generating a complex interplay, which is tightly modulated by miRNAs.

## 2. MicroRNA as Regulators of Immune Homeostasis in Autoimmune Diabetes

Among the broad range of functions in which miRNAs are involved, there is growing evidence that miRNAs are crucial modulators of immune cell functions, thus representing major players in the regulation of immune homeostasis. In particular, miRNAs are associated with many facets of immune responses such as development, activation, and differentiation. In order to uncover the role of miRNAs in murine immune system, several studies depleted miRNAs in specific immune cells by deleting key enzymes involved in miRNA biogenesis. For example, Lck-Cre-mediated ablation of Dicer during thymocyte development compromised the survival of *αβ*-lineage cells, whereas the number of *γδ* expressing thymocytes was not affected [7]. Remarkably, the CD4/CD8 lineage commitment appeared unaltered in the absence of Dicer. Conditional deletion of Dicer in CD4+ T cells at a later time point resulted in reduced cell number due to both increased cell death and impaired proliferation. As for T helper (Th) cell polarization, Dicer-deficient CD4+ T cells failed to differentiate into Th2 phenotype due to their increased polarity to Th1 effector cells and to the inability to block interferon-*γ* (INF-*γ*) production [8]. Of note, similar results were obtained upon Drosha or Argonaute deletion in T cells [9, 10]. Given miRNAs multiple roles in T cells, it is not surprising that these short noncoding RNA molecules regulate also humoral immunity by fine tuning B cells differentiation and activation. Thus, ablation of Dicer in early B cell progenitors results in a substantial arrest at the pro-B to pre-B cell transition [11], while ablation of Dicer in antigen activated B cells, that is, at a later time point, results in a defective production of high affinity class-switched antibodies with an impaired formation of germinal centre B cells, long-lived plasma cells, and memory B cells [12]. Taken together, these studies show how global miRNAs deletion has detrimental effects on T and B cells development and function; moreover, mice with miRNA-deficient CD4+ T cells are prone to develop immune pathology as they age, suggesting a role for miRNAs in the maintenance of immune homeostasis [7]. Among immune cells with regulatory activity, CD4+ regulatory T (Treg) cells play an essential role in immune homeostasis and self-tolerance. Treg cells are characterized by high expression of the IL-2R*α* chain (CD25) and the transcription factor forkhead box P3 (FOXP3), which serves as a lineage specification factor. The role of Treg in autoimmunity is highlighted by patients with IPEX (Immune-dysregulation Polyendocrinopathy Enteropathy X-linked syndrome), in which absence of Treg cells results in an enhanced susceptibility to autoimmune diseases including type 1 diabetes [13].

Specific ablation of miRNAs in Treg cells secondary to Dicer or Drosha deletion resulted in an early fatal onset of autoimmunity similar to what has been previously observed in FOXP3-deficient mice [9]. Indeed, miRNA-deficient Treg cells show decreased levels of FOXP3 together with altered differentiation and function [14–16]. However, since lack of Dicer and Drosha interferes with the generation, not only of canonical miRNAs, but also of other small RNA species, Jeker et al. generated mice with Treg cells lacking Dgcr8, RNA-binding protein required in the processing of canonical miRNAs. Interestingly, mice lacking canonical miRNAs showed a similar phenotype to that of Dicer-deficient mice, thus demonstrating that miRNAs are essential for normal FOXP3 expression and suppressive Treg function and suggesting a high degree of dependence on miRNAs-mediated regulation for normal Treg development and function [15]. Several studies have also begun to uncover the role of single miRNAs in immune cells. Studies by Cobb et al. in a murine model uncovered a characteristic set of miRNAs, which are enriched in Treg with respect to naïve CD4 T cells; these include miR-223, miR-146, miR-155, miR-21, and miR-24 [17]. Interestingly, miR-155 is of particular interest because of the FOXP3 mediated regulation of the intronic region of B cell integration cluster (Bic), which encodes the gene for miR-155 [18]. miR-155 deficient mice showed reduced number of thymus-derived and peripherally induced Treg cells [19]; in contrast, Treg suppressive function was unaffected both* in vitro* and* in vivo* in these mice [20]. Of note, miR-155 guarantees Treg cell homeostasis by targeting suppressor of cytokine signal 1 (SOCS1), a negative regulator of IL-2 pathway, thereby increasing the sensitivity of these cells to their main growth factor with a crucial role in Treg cells development [19].

In addition to miR-155, miR-146a was identified to be prevalently expressed in Treg cells and important for their function. Lu et al. have shown that miR-146a deficiency, limited to FOXP3+ Treg cells using a mixed bone marrow chimera approach, resulted in IFN-*γ*- and Th1-mediated disorders, similar to the alterations observed in mice bearing Treg cell-specific Dicer or Drosha deficiency [21]. This phenomenon can be explained by the direct targeting of miR-146a to the signal transducer and activator of transcription 1 (STAT1), a key transcription factor in IFN-*γ* response and in Th1 differentiation [21].

It is becoming increasingly evident that miRNAs modulate several pathways involved in immune cells homeostasis and that miRNA alterations can lead to specific dysfunctions that could favor the development of autoimmune diseases including T1D.

Jeker et al. have shown that miR-10a is preferentially expressed in mouse thymus-derived Treg cells, although it does not seem to directly regulate FOXP3 or other factors involved in Treg homeostasis. Interestingly, miR-10a expression is lower in Treg of nonobese diabetic mice (NOD), a spontaneous murine model of autoimmune diabetes, whereas miR-10a levels are higher in autoimmunity-resistant C57BL/6 mouse strain [22]. Moreover, Takahashi et al. showed that miR-10a could be induced by retinoic acid and by TGF-*β* in inducible Treg cells [23]. Indeed, they showed that miR-10a attenuated phenotypic conversion of inducible Treg cells to follicular helper T cells by simultaneously targeting the transcriptional repressor Bcl-6 and corepressor Ncor2 [23]. Thus, miR-10a is a candidate player involved in the maintenance of Treg cell specific phenotype by targeting factors that could lead to conversion to other cell fates.

As for the potential protective role of miRNAs in autoimmune diabetes, Berry et al. showed a connection between miR-34a and diabetes protection [24], reporting an impaired B cell lymphopoiesis in diabetes-resistant NOD.B10 Idd9.3 mice, congenic for insulin-dependent diabetes (Idd) Idd9.3 locus. Interestingly, they showed that miR-34a was significantly higher in B cell progenitors and in marginal zone B cells from NOD.B10 Idd9.3 mice versus wild type NOD mice. Furthermore, miR-34a expression in these cell populations inversely correlated with levels of Foxp1, an essential regulator of B cell lymphopoiesis, which is directly repressed by miR-34a. Moreover, mature B cells from NOD.B10 Idd9.3 mice were unable to prime islet-specific CD4+ T cells* in vitro*, which may contribute to T1D protection in NOD.B10 Idd9.3 mice [24].

Recently published studies have also reported miRNA specific signatures in peripheral blood immune cells from type 1 diabetic patients. Interestingly, analysis of miRNA performed by Hezova et al. in Tregs from normal and from type 1 diabetic subjects showed an increased expression of miR-510 and a decreased expression of miR-342 and miR-191 [25]. Salas-Pérez and colleagues showed that miR-21a and miR-93 are reduced in peripheral blood mononuclear cells (PBMC) of patients with T1D, likely as a consequence of chronic hyperglycemia since cultures of PBMC from nondiabetic subjects downregulated miR-21a expression levels when cultured in the presence of high glucose [3]. In contrast, glucose levels in culture media did not affect miR-93 expression in PBMC and bioinformatic analysis of miR-93 predicted targets showed STAT3 (a protein with a crucial role in immune responses) as potential target gene [3].

Yang et al. compared miRNA expression profiles of PBMC from newly diagnosed T1D patients versus nondiabetic individuals, identifying miR-146a as the most downregulated miRNA in PBMC of T1D patients; this downregulation was independent of hyperglycemia and of disease duration, while it was associated with high glutamic acid decarboxylase autoantibodies (GADA) titers [26]. The association between miRNAs expression and severity of autoimmune response in T1D was also previously analyzed by our group. Indeed, we analyzed miRNAs expression in PBMC from patients with T1D showing an increased expression of miR-326, which was positively correlated with the presence of diabetes-associated autoantibodies [4]. Interestingly, bioinformatic target genes analysis revealed vitamin D3 receptor (VDR) and ETS1 as putative miR-326 target genes [4]. Both are reported to be involved in the regulation of immune cell homeostasis and thus it is possible to speculate that an alteration of miR-326 may regulate islet autoimmunity. Interestingly, miR-326 has also been found to be upregulated in peripheral blood leukocytes (PBL) of patients with relapsing remitting multiple sclerosis when compared to age-matched controls [27]. Moreover, PBLs from patients with relapsing multiple sclerosis had significantly higher miR-326 expression than those from patients with remitting multiple sclerosis, suggesting an association between miRNAs expression and severity of autoimmune response.

Taken collectively these findings demonstrate how miRNA regulation of lymphocyte biology can be crucial for the maintenance of self-tolerance. On the other hand, there is increasing evidence suggesting a contribution of the innate immune system to the breakdown of tolerance in several autoimmune diseases. The innate immune system ensures a first line of defence against foreign pathogens and is mediated mainly by macrophages, granulocytes, dendritic cells, and natural killer cells. Recently, several studies are shedding light on the role of the innate immune system in T1D development [28–30]. For example, new evidence demonstrated that dendritic cells play a pivotal role in activating naïve T cells with islet-specific antigen autoreactive capacity. Indeed, it has been suggested that diabetic NOD mice dendritic cells have increased potential to activate T cells compared to those derived from nondiabetic mice, mainly through augmented IL-12 production and costimulatory molecule expression [31, 32], thus demonstrating an active role of dendritic cells in the induction or exacerbation of autoimmune responses.

Interestingly, terminally differentiated and short-lived cells such as neutrophils are also likely to play a role in autoimmune diabetes. Valle et al. have observed a reduced number of circulating neutrophils in patients with established diabetes as well as in normoglycemic subjects with high risk for T1D development (nondiabetic first-degree relative of T1D patients) with respect to nondiabetic subjects [33], while an increased infiltration of neutrophils has been observed in the pancreatic exocrine tissue of T1D donors. The authors concluded that neutrophils may play a role in the initiation and progression of the disease.

Additional components of innate immune cells such as *γδ* T-lymphocytes have been linked to diabetes. Indeed, Markle et al. using an adaptive transfer model have shown the detrimental role of IL-17-producing *γδ* T cells in diabetes development [34].

Another essential element of the innate immune system is the Toll Like Receptor (TLR) system, which consists of a group of transmembrane proteins expressed in immune and in nonimmune cells, whose role is to recognize components of foreign pathogens. TLRs have been associated with T1D development in mouse and in human studies [35, 36], thus underlining the importance of innate immune system in diabetes etiology.

Emerging data have identified miRNAs as important regulators of development and function of innate immune components in addition to the aforementioned adaptive immune cells. For example, Cebpa-Cre driven deletion of Dicer compromises definitive maturation of neutrophils from myeloid precursors [37]. Moreover, studies focusing on the effect of Dicer ablation in skin-draining lymph nodes migratory dendritic cell subset, namely, Langerhans cells, showed a disturbed expression of surface molecules and reduced antigen presentation capacities to CD4+ T cells, increased turnover, reduced half-lives, and increased rates of apoptosis [38].

Interestingly, as already mentioned, Lck-Cre-mediated ablation of Dicer during thymocyte development does not affect *γδ* T cell number; in contrast there is a substantial increase of *γδ* T cells in the double negative thymic compartment [7]. Lastly, also TLR signalling pathways are deeply regulated by miRNAs [39].

It is becoming increasingly evident how miRNAs modulate immune cell function and how miRNA dysregulation in these cells can lead to immune pathology. However, further studies are needed to fully elucidate the role of single miRNA as well as of groups of specific miRNAs in the regulation of the immune response in health and in disease.

## 3. MicroRNAs: Active Participants in the Immune-Mediated Beta-Cell Damage

Pancreatic islet inflammation and beta-cell damage represent a hallmark of both type 1 and type 2 diabetes. Although the etiology differs between the two forms of diabetes, immune cell infiltrates have been observed in both of them [40]. Beta-cell dysfunction and damage can be in part attributed to the unfavorable environment mainly characterized by detrimental milieu of proinflammatory cytokines, which represents a common phenomenon occurring in type 1 and type 2 diabetes.

Beta-cells are very sensitive to diabetes-associated inflammatory mediators and in turn activate a series of molecular mechanisms which can lead to (i) beta-cell dysfunction (e.g., reduced insulin secretion or insulin content); (ii) beta-cell apoptosis; (iii) exacerbation of inflammatory phenomena through the secretion of chemokines which lead to an increased immune cell infiltrate. The molecular mechanisms involved in these types of responses are tightly regulated by miRNAs.

One of the first studies aimed at elucidating the effects of an inflammatory environment on beta-cell miRNA expression has been published in 2010 by Roggli and colleagues [2], who analyzed the effects of a combination of cytokines on miRNA expression profiles in beta-cells and their putative link to beta-cell function and/or survival. To this end, authors exposed beta-cell line MIN6 cells (mouse insulinoma beta-cell line 6) for 24 hours to cytokines typically secreted by infiltrating immune cells: IL-1*β* or a combination of IL-1*β*, TNF-*α*, and IFN-*γ*. The evaluation of miRNA expression profiles through microarray technology revealed three differentially expressed miRNAs. Specifically, miR-21, miR-34a, and miR-146a expression levels were increased both by IL-1*β* and by the cytokine mix. Of particular interest, IL-1*β* alone was revealed as the strongest inducer of miR-21 and miR-146a expression, although these miRNAs were also upregulated by TNF-*α* but not by IFN-*γ*. The expression of miR-34a was equally stimulated by IL-1*β* and TNF-*α* while IFN-*γ* did not show any effect on its expression levels [2]. Importantly, similar results, characterized by an increased expression of miR-21, miR-146a, and miR-34a, were obtained upon exposure of cultured human islets to IL-1*β*. The same expression pattern was also clearly observed in pancreatic islets isolated from NOD mice at 8 and 13 weeks of age versus 4-week-old mice. Of note, 4-week-old NOD mouse pancreatic islets did not show any sign of infiltration, indicating the relevance of infiltrating immune cells in the secretion of cytokines, which in turn modulate the expression of these miRNAs. The exposure of MIN6 cells to IL-1*β* induced a reduction of insulin gene activity and of proinsulin mRNA content; however, overexpression of miR-21 and miR-146a in MIN6 cells did not induce such effects, while a slight reduction of insulin gene activity and proinsulin mRNA was observed upon miR-34a overexpression [2]. Conversely, it was shown that miR-34a and miR-21 had major effects on glucose stimulated insulin secretion (GSIS) through the regulation of VAMP2 expression, a molecule involved in insulin granule exocytosis, and of Rab3a (GTPase) and that this effect can be mimicked by a weak and short exposure to cytokines. In support of this, it has been demonstrated that, in MIN6 cells, overexpression of miR-34a and of miR-21, but not of miR-146a, leads to an impaired GSIS, while the inhibition of their activity during IL-1*β* exposure improved GSIS.

Whereas short exposure to cytokines leads to an impaired insulin secretory machinery, a prolonged cytokine exposure leads to increased apoptotic cell death. Interestingly, it was shown that this phenomenon might be partially mediated by miRNAs. Indeed, a lower cell death rate was observed by blocking miR-34a or miR-146a activity, while miR-21 knockdown did not show such effect and, conversely, promoted apoptosis. These data indicate a role for these miRNAs in cytokine-mediated cell death secondary to prolonged cytokine exposure and established a link between cytokines and miRNAs in beta-cells.

Although miR-34a and miR-146a have a deleterious effect on beta-cell survival by promoting apoptosis, the role of miR-21 has not yet been clearly elucidated. The function of miR-21 relies on the effect of various cytokines and/or growth factors via NF-*κ*B pathway [41]. A recent study pointed out a specific role for miR-21 strengthening its role in protecting beta-cells from cytokines induced apoptosis. Indeed, in this study the authors characterized the molecular mechanism, which leads to the expression of miR-21 and to protection of beta-cells from apoptosis [42]. They specifically identified a relationship between miR-21 and PDCD4 (programmed cell death protein 4). PDCD4 has been previously characterized as a pivotal factor in a number of apoptotic pathways and its overexpression in the beta-cell line betaTC1-6 increased susceptibility to cytokine-mediated cell death. Indeed, it was demonstrated that PDCD4 deficiency rendered mouse islets resistant to cytokine-induced apoptosis, thus highlighting a deleterious effect of PDCD4 in beta-cell survival. To elucidate the molecular link between PDCD4 and miR-21, the authors showed that miR-21 targets PDCD4 and that their expression levels are inversely correlated during exposure of betaTC1-6 to proinflammatory cytokines. Moreover, they demonstrated that inhibition of NF-*κ*B decreased the miR-21 expression levels leading to a significant increase of PDCD4 mRNA and to an increased cell death. In light of such data it is possible to hypothesize that NF-*κ*B exerts its antiapoptotic effect through the induction of miR-21 which in turn negatively regulates the expression levels of cell death inducer PDCD4, thus leading to a protective response during cytokines exposure of beta-cells. As a matter of fact, this study is perfectly in line with Roggli et al., who demonstrated that inhibition of miR-21 promoted apoptosis in beta-cells treated with proinflammatory cytokines.

Another example of miRNA contribution to the progression of insulitis has been reported in 2012 by Roggli et al., who examined miRNA expression profiles in pancreatic islets of NOD mice at different disease stages. Specifically, they analyzed pancreatic islets isolated from prediabetic NOD mice at 4 weeks, 8 weeks, and 13 weeks of age. The results showed a differential expression of several miRNAs and, among these, they focused on miR-29 family, composed of miR-29a, miR-29b, and miR-29c [31]. They observed a progressive increase of miR-29 expression levels during insulitis progression in NOD mice with a maximum upregulation at 13 weeks of age. A similar expression pattern was observed in MIN6 cells exposed to a proinflammatory cytokines mix; this effect was observed also in mouse islets and in human islets, thus highlighting a conserved effect on the expression of miR-29 family members following beta-cell exposure to cytokines [43]. Of note, upregulation of miR-29 family members induced a reduction of insulin mRNA content and a decreased GSIS, mainly exerted by miR-29 targeting and negative regulation of Onecut2 (a transcriptional repressor of granuphilin-4, an inhibitor of insulin granules exocytosis). A specific role for miR-29 in the exacerbation of cytokine-induced apoptosis was also identified. This effect was mainly mediated by the downregulation of Mcl1, an antiapoptotic protein, which has been demonstrated to be a specific target of miR-29 family members [43].

The role of miRNAs has also been evaluated in other inflammatory stimuli. A recent study by Bravo-Egana and colleagues evaluated miRNAs expression profiles in rat islets transplanted under the kidney capsule of syngeneic recipients [44]. Indeed, inflammation plays a key role in islet engraftment and survival after transplantation. In the posttransplant period, the graft is exposed to a series of inflammatory stimuli characterized by chemokine secretion, host tissue factor, and/or macrophages activation, which lead to a detrimental graft environment. In this context a pattern of 31 miRNAs with altered expression was identified: 26 of them were upregulated and 5 downregulated. This dataset was compared with miRNA expression profiles of rat islets exposed* in vitro* to a mixture of IL-1*β*, TNF-*α*, and IFN-*γ*; eight miRNAs correlated between the two conditions; specifically, miR-21, miR-98, miR-27a, miR-143, let-7d, miR-126, and miR-22 were upregulated, while miR-129 was downregulated upon inflammatory environment exposure. Bioinformatic analysis of predicted target genes, coupled to a previously published microarray dataset of differentially expressed mRNAs during proinflammatory stimuli of beta-cell [45], revealed that most of the differentially expressed miRNAs putatively regulate the expression of genes involved in the control of insulin signal transduction, islets development and function, and insulin secretion.

The importance of miRNAs in cytokine-mediated beta-cell damage has also been pointed out in a study focused on the evaluation of overall miRNAs contribution in immune-mediated cell distress [46]. In this study the role of miRNAs was analyzed in a mouse model of autoimmune diabetes, which is initially triggered by multiple low doses of streptozotocin (MLDS). Immune-mediated beta-cell death is a hallmark of MLDS. Whereas a single high dose of streptozotocin mainly induces beta-cell death via a direct toxic effect, MLDS induces a major increase in the production of IFN-*γ*, IL-1*β*, and TNF-*α*, which lead to beta-cell death and dysfunction. In this study, the incidence of diabetes in wild type mice versus beta-cell specific Dicer knockout mice (DICER-RIP-Cre KO miRNA-deficient beta-cell) exposed to MLDS has been evaluated. The results showed that the incidence of MLDS-induced diabetes was higher in DICER-RIP-Cre KO versus WT mice, indicating that the lack of miRNAs in beta-cells enhances the susceptibility to MLDS-induced diabetes and accelerates and exacerbates diabetes onset [46]. However, in the explanation of this phenomenon it should be taken into account the fact that the elimination of miRNAs biogenesis machinery can induce severe beta-cell dysfunction and diabetes, per se, through retarded and incomplete beta-cell maturation.

Recently, it has been reported that the differentiation status of beta-cells is a fundamental prerogative for their susceptibility to deleterious effects of proinflammatory cytokines. As a matter of fact, mature beta-cells are sensitized to the effects of IL-1*β*, while an immature phenotype confers protection [47]. Indeed, miRNAs expression profiles analyzed in INSr*αβ* cells (a subclone of INS-1 beta-cell line) exposed to IL-1*β*, with or without a mature phenotype (conferred by induction of PDX1 expression), revealed a differential expression of miRNAs dependent on the cell maturation status. Specifically, the exposure of mature INSr*αβ* cells to IL-1*β* induced a differential expression of miR-375 and miR-194 (upregulation of miR-375 and downregulation of miR-194), whereas this effect was lost in the immature INSr*αβ* cells, highlighting the importance of phenotype in response to stress stimuli [48].

The concept of beta-cell phenotype loss as a mechanism of self-protection from metabolic and/or inflammatory insults has been recently introduced by Domenico Accili's group. Indeed, Talchai and colleagues found strong evidence of beta-cell dedifferentiation in several murine models of diabetes, describing a regression of beta-cell to an endocrine progenitor-like phenotype, pointing out an advantage to beta-cell in adopting a dedifferentiated fate, which may facilitate their survival [49]. In this context, miRNAs may play major roles in the maintenance of beta-cell phenotype, thus representing candidate regulators of dedifferentiated fate acquisition. Indeed miRNAs are involved in the control of beta-cell phenotype through a tight regulation of those genes necessary for fate transition and/or specification [50]. In consequence of their possible alteration, miRNAs may be responsible for beta-cell phenotype loss as well as sensitization to several cytokines or other types of inflammatory stressors in type 1 or type 2 diabetes.

**Figure 1:**
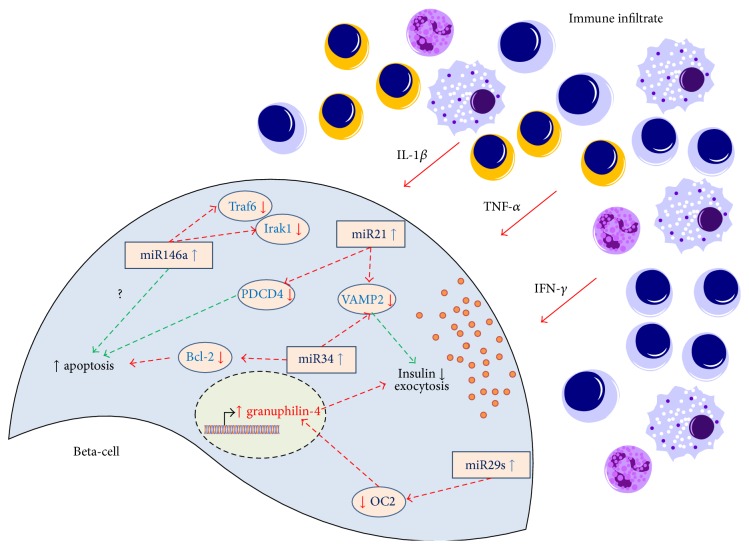
MicroRNAs as major players in immune-mediated beta-cell damage in autoimmune diabetes. The figure reports a representing scheme of microRNAs (miRNA) response induced by infiltrating immune cells (mainly CD4+ and CD8+ T-lymphocytes, B-lymphocytes, and macrophages) and secreted cytokines. The secretion of IL-1*β*, TNF-*α*, and IFN-*γ* induces changes in expression of several miRNAs. Specifically, upregulation of miR-146a, miR-34, miR-21, and miR-29s is shown. Each miRNA targeting specific genes (with consequent inhibition of their expression) is reported as a red dotted arrow, while the activation of a specific mechanism is represented as green dotted arrow. Upregulation of miR-146a induces the activation of apoptosis pathway through a not yet understood mechanism, while inhibiting the expression of Traf6 and Irak1 (NF-*κ*B pathway). Upregulation of miR-34 leads to reduction of antiapoptotic gene Bcl-2 and of VAMP2, a molecule involved in the fusion of insulin granules to the plasma membrane. The increased expression of miR-21 leads to a partial protection from apoptosis through the inhibition of cell death inducer PDCD4. It also decreases expression levels of VAMP2, thus blocking insulin secretion. Finally, upregulation of miR-29s (miR-29a-b-c) leads to reduction of OC2 (Onecut2), a transcriptional repressor of granuphilin-4 (inhibitor of insulin secretion), thus leading to its upregulation and subsequently to a reduced insulin exocytosis.

Altogether, these studies demonstrate that miRNAs are pivotal players in the response of beta-cell during immune-mediated damage. Indeed, miRNAs actively modulate several different pathways in response to cytokines stimuli, as depicted in Figure 1. The presence of infiltrating cells within pancreatic islets generates a cytokines milieu principally composed of IL-1*β*, TNF-*α*, and IFN-*γ*. These cytokines induce detrimental effects in beta-cells also through the modulation of several miRNAs. Therefore, the upregulation of miR-146a, miR-34, and miR-29s induces beta-cell apoptosis through different mechanism, while the upregulation of miR-21 seems to confer a protective effect. miR-34, miR-29s, and miR-21 upregulation induced also the alteration of insulin granules exocytosis through the inhibition of several genes involved in the regulation of glucose stimulated insulin secretion (e.g., VAMP2, OC2). Furthermore, recent lines of evidence reported that cells are able to communicate using secreted miRNAs contained within budding microvesicles. It is not unlikely that both beta-cells and immune infiltrating cells are able to exchange information via microvesicles containing miRNAs and that some of them may also confer detrimental effects both in endocrine and immune cell components.

## 4. Secreted MicroRNAs: Messengers in the Dialogue between Pancreatic Islets and the Immune System

While the majority of miRNAs have been found inside cells, several miRNAs have also been detected in biological fluids such as plasma, serum, urine, saliva, semen, and breast milk [51, 52] usually complexed with ribonucleoproteins (such as Argonaute 2) or lipoproteins (HDL, LDL) or inside membrane-derived vesicles [53–56]. Such complexes confer protection from RNAse degradation with consequent high degree of stability and measurability. However, the origin of circulating miRNAs is still unclear although two main hypotheses have been raised: the first argues that miRNAs could be passively released into the circulation during tissue injury, remaining stably complexed to ribonucleoproteins in the extracellular environment [57, 58], whereas the second supports the active and regulated secretion of endogenous miRNAs within microvesicles (e.g., exosomes), which are released into the extracellular environment by different cell types [59]. Several reports proposed that regulated secretion of miRNA-containing microvesicles may play an important role in cell-to-cell communication, both in physiological and in pathological conditions. There are three different types of microvesicles containing extracellular miRNAs: apoptotic bodies, shedding vesicles, and exosomes [60]. The latter are vesicles of 40–100 nm diameter, which are formed via the inward budding of plasma membrane into multivesicular bodies within endosomes. In response to extracellular stimuli, in both physiological and pathological conditions, they are released into the extracellular compartment upon fusion of endosomes with the plasma membrane of the recipient cell [61, 62]. Exosomes are produced by a wide variety of cell types including reticulocytes, neurons, and epithelial and tumor cells, as suggested by* in vitro* studies [55, 61, 63–65], and appear to contribute to different biological functions including immune response, antigen presentation, and protein and nucleic acids transport [55, 66–68]. It is also possible to hypothesize that specific miRNAs can be selectively secreted from cells, packaged into appropriate carriers to be transported to target cells (or tissues), and released into recipient cells, for example, through the fusion of exosomes with the plasma membrane or by binding to specific receptors, in order to regulate gene expression [69–71]. It remains unclear whether circulating miRNAs are secreted in a specific and signal-dependent manner [55, 71–74]. Indeed, gaining insight into the relationship between circulating and tissue miRNAs will certainly contribute to the understanding of the origin of circulating miRNAs. To date, many studies in oncology have reported a similar trend of expression between circulating miRNAs and tissue miRNAs [59, 75, 76]. In contrast, several authors have also described opposite differential expression profiles of miRNAs transported in vesicles compared to parent cells, suggesting that some miRNAs may be transcribed only to be exported or that their secretion is tightly regulated [55, 72, 74].

In light of these studies, it is not unlikely that virtually all cells may be able to secrete miRNAs in a specific and controlled fashion, in order to regulate gene expression also in distant tissue sites. Recently, it has been suggested that both islet beta-cells and immune cells are capable of miRNAs secretion and that this phenomenon may represent a mechanism involved in functional defects both at islet and at immune system level. Indeed, a recent study reported the analysis of miRNA content in microvesicles released by several beta-cell lines and primary pancreatic islets and tested the biological relevance of miRNA transfer among beta-cells. Analysis of the global profile of miRNAs in released microvesicles revealed that it did not reflect that of the parent cells. Importantly, some miRNAs poorly expressed in parent cells were abundant in vesicles, suggesting a preferential release of a subset of miRNAs. It was also shown that the expression levels of several miRNAs released into the medium changed upon exposure of beta-cells to cytokines or to palmitate. Indeed, the incubation of naïve beta-cells with miRNAs-containing microvesicles from donor cells treated with cytokines does not affect the secretory functions of recipient cells but results in an increase in apoptosis. Taken together, these data suggest that the cytokine milieu derived from immune cells infiltrating islets can negatively influence miRNAs secretion from beta-cells, thereby leading to islet dysfunction [77].

In another study, human islets released biological active extracellular vesicles, carrying specific miRNAs (such as miR-27b, miR-126, miR-130, and miR-296) involved in beta-cell function, insulin secretion, and angiogenesis and expressing surface molecules as well as islet-specific proteins (e.g., insulin, C-peptide). Interestingly, microvesicles content could be transferred to endothelial cells, resulting in the induction of insulin mRNA expression and protection from apoptosis and angiogenesis. These data demonstrated that human islets are able to release biological active extracellular vesicles and to transfer beta-cell specific proteins, mRNAs, and miRNAs to endothelial cells, inducing a proangiogenic and antiapoptotic cell phenotype [78].

Given the secretion of miRNA-containing vesicles from parent cells into the extracellular space, it is also possible to speculate that this phenomenon may be mirrored in serum or in plasma. Indeed, several studies have been performed on the evaluation of circulating miRNA expression profiles in serum or plasma of T1D patients, aiming at elucidating the potential role of circulating miRNAs in diabetes.

To this aim, we recently compared the expression profile of circulating miRNAs of 20 T1D patients with that of control subjects showing that 64/206 miRNAs detected were differentially expressed in sera of patients with T1D. Interestingly, these miRNAs are implicated in the regulation of beta-cell function and cell belonging to the immune system [79]. Another study by Nielsen and colleagues compared the expression profile of miRNAs in serum of children with T1D and control subjects and identified 12 miRNAs (including some involved in apoptosis of beta-cells) that were overexpressed in children with T1D (miR-152, miR-30a-5p, miR-181a, miR-24, miR-148a, miR-210, miR-27a, miR-29a, miR-26a, miR-27b, miR-25, and miR200a). Moreover, circulating levels of miR-25 were correlated to the residual beta-cell function (assessed by C-peptide measurement) and with adequate glycemic control (assessed by the determination of glycated hemoglobin) three months from T1D onset [80].

Experimental lines of evidence associated circulating miRNAs with the development of immune-mediated diseases [81]. Transfer of molecules during immune cells interactions has been widely reported [82] and, to date, the role of exosomes in such phenomenon has been investigated by several authors [83, 84]. Two previous studies clearly demonstrated that transformed Epstein-Barr virus B-lymphocytes and dendritic cells were able to secrete exosomes with the capacity to present MHC-peptide complexes to specific T cells, suggesting a key role for exosomes in intercellular communication in immune system [85, 86]. More recently, several data indicated an exosomes-mediated transfer of miRNA between T cells and antigen presenting cells during antigen recognition and a capacity of transferred miRNAs to regulate gene expression in recipient cells, during immune synapse formation [73, 82].

In a recent study, Mittelbrunn et al. showed that exosomes isolated from T cells, B cells, and dendritic cells presented a unique miRNAs expression profile when compared to their parent cells. In particular, several miRNAs (such as miR-760, miR-632, miR-654-5p, and miR-671-5p) were significantly more expressed in exosomes from all cell types, like miR-335, which were found only in exosomes derived from dendritic cells; conversely, several miRNAs (such as miR-101, miR-32) were found to be more abundant in cells than in exosomes. Interestingly, these exosomes were able to mediate the antigen-driven unidirectional transfer of functional miRNAs (i.e., able to modulate gene expression in recipient cells) from T cells to antigen presenting cells during immune synapse formation. These data suggest that miRNAs are transferred during immune synapse formation, thus mediating several functions in recipient cells [73].

Evidence of the effects of circulating miRNAs in the modulation of immune system in diabetes was reported by Salama and colleagues [87] who showed that pancreatic beta-cell-derived miRNAs induce proinflammatory (TNF-*α*, IFN-*α*, IL-12, and IL-6) or suppressive (IL-10) cytokine secretion by mouse dendritic cells. Of note, miR-29b, by interacting with TLR7, stimulates dendritic cells to produce several cytokines and chemokines. Moreover, in a murine model of adoptive transfer of autoimmune diabetes, antigen-specific T cell responses and disease incidence were decreased by the systemic delivery of miR-29b, while* in vitro*, exosomes derived from beta-cells presented specific miRNA expression profiles, including miR-29b. These exosomes were shown to induce cytokine secretion (including TNF-*α*) from splenocytes isolated from NOD mice, while, in the presence of miR-29b, TNF-*α* secretion was impaired. On the basis of these results, it was suggested that beta-cell specific miRNAs, such as miR-29b, may modulate autoimmune response by recruiting innate immune cells through receptor-ligand interactions [87].

The hypothesis that miRNAs could mediate intercellular communication is fascinating. However further studies are still needed to fully demonstrate this hypothesis and several questions remain open. For example, it remains to be elucidated how extensively the process of cell-to-cell communication via extracellular miRNAs occurs* in vivo*. Finally, it has not been established in which form miRNAs are present in vesicles (i.e., as mature or pre-miRNAs) [58].

## 5. Concluding Remarks

An increasing number of studies have uncovered the role of miRNAs in autoimmune diabetes, both at pancreatic islet and at immune system level. Moreover, growing evidence indicates that miRNAs represent major players in the dialogue between beta-cells and immune system, regulating several aspects of their function and survival (Figure 1).

Interestingly, miRNAs have been found to be stably present in the extracellular environment, thus suggesting a possible new emerging role for miRNAs in cell-to-cell communication, in addition to their better-understood intracellular role as negative regulators of gene expression. Given the impact of miRNAs on beta-cells and immune cells in physiologic and disease conditions, secreted miRNAs may represent an important mechanism of islets-immune cell dialogue in autoimmune diabetes. Furthermore, being stable in extracellular fluids such as serum and plasma, miRNAs represent promising biomarkers for disease diagnostics and staging. Further studies aiming at clarifying miRNAs expression profiles in pancreatic islets or more specifically in pancreatic islets and in islet-infiltrating immune cells will shed light on these newly identified phenomena.
